# Accentuating and Opposing Factors Leading to Development of Thoracic Aortic Aneurysms Not Due to Genetic or Inherited Conditions

**DOI:** 10.3389/fcvm.2015.00021

**Published:** 2015-05-26

**Authors:** Simon W. Rabkin

**Affiliations:** ^1^Division of Cardiology, Department of Medicine, University of British Columbia, Vancouver, BC, Canada

**Keywords:** thoracic aortic aneurysm, pathophysiology

## Abstract

Understanding and unraveling the pathophysiology of thoracic aortic aneurysm (TAA), a vascular disease with a potentially high-mortality rate, is one of the next frontiers in vascular biology. The processes leading to the formation of TAA, of unknown cause, so-called degenerative TAA, are complex. This review advances the concept of promoters and inhibitors of the development of degenerative TAA. Promoters of TAA development include age, blood pressure elevation, increased pulse pressure, neurohumeral factors increasing blood pressure, inflammation specifically IFN-γ, IL-1 β, IL-6, TNF-α, and S100 A12; the coagulation system specifically plasmin, platelets, and thrombin as well as matrix metalloproteinases (MMPs). SMAD-2 signaling and specific microRNAs modulate TAA development. The major inhibitors or factors opposing TAA development are the constituents of the aortic wall (elastic lamellae, collagen, fibulins, fibronectin, proteoglycans, and vascular smooth muscle cells), which maintain normal aortic dimensions in the face of aortic wall stress, specific tissue MMP inhibitors, plasminogen activator inhibitor-1, protease nexin-1, and Syndecans. Increases in promoters and reductions in inhibitors expand the thoracic aorta leading to TAA formation.

The potentially high-mortality rate of thoracic aortic aneurysm (TAA) (1, 2), highlights the need for a greater understanding of its pathophysiology so as to prevent TAA expansion and rupture. Although TAA can be due to a variety of different genetic or inherited conditions, the majority of TAA cases, are usually ascribed to “degenerative” factors (3). The development of idiopathic or “degenerative” forms of TAA is complex and poorly understood. Degenerative TAAs develop during processes in which factors leading to aneurysm formation overcome factors, which retard TAA development. The objective of this review is to discuss the putative factors that accentuate and those that retard the development of degenerative TAA. Because the structure and embryologic origins of the cellular composition of the thoracic aorta are different from the abdominal aorta, this review will rely mainly on data from TAA and not from studies of the abdominal aorta aneurysms (AAAs).

## Normal Thoracic Aortic Structure

The major component of the media of the thoracic aorta is dozens of layers of “lamellar units” consisting’s of two elastic lamellae and intervening tissue that are oriented in concentric layers around the lumen (4, 5). The extracellular matrix of the lamellar unit is complex and “consists of a wide range of components, every one of which has a highly specific spatial relation to the others” (5). The elastic lamellae are closely associated with collagen fibers (types I, III, and V collagen) and fibronectin (5, 6). The lamellae are interconnected by a network of small elastic and collagen fibers as well as proteoglycans (6). Smooth muscle cells are also in contact with fibrillin-1- and type VI collagen-containing as well as bundles of microfibrils (oxytalan fibers) (5). Smooth muscle cells of the media have a basal lamina-like layer connecting them to each other as well as to oxytalan fibers (5). Proteoglycans mainly collagen-associated dermatan sulfate proteoglycan, cell-associated heparin sulfate proteoglycan, and interstitial chondroitin sulfate proteoglycan are other components of the vascular wall (5).

The constituents of the arterial wall are responsible for its mechanical properties and the ability of the vessel to prevent or limit permanent deformation. Elastic fibers comprise elastin, microfibrils that include the fibulins, fibrillins, and microfibril-associated glycoproteins, restore the vessel wall to its resting conditions after systolic expansion (5, 7). Collagen fibers and the microarchitecture that connect them prevent “mechanical failure” of the vessel under the constant loading of arterial pressure and its further increase during systole (7).

## Structural Changes in the Aortic Wall in Thoracic Aortic Aneurysm

The aortic wall in TAA shows fragmentation of elastin and collagen fibers (6, 8) and accumulation of glycosaminoglycans in the media (9). There is a disorganization and breakdown of the elastin network and its interconnection with collagen network and other components of the aortic wall (6). Fibronectin distribution is heterogeneous in the aneurysmal aortic wall with areas of “acellular cystic medial degeneration,” which are devoid of fibronectin and consist of clumps of compact fibronectin around shrunken smooth muscle cells (6). Fibulin-5, an extracellular protein that regulates elastic fiber assembly, is reduced in TAA especially in TAAs that undergo aortic dissection (10, 11).

While some investigators contend that the amount of collagen in TAA is increased (12), other investigators found that the type of collagen is altered specifically type I and III collagens are significantly decreased while collagens alpha1(XI) and V are significantly increased (13). This shift in collagen type may alter aortic structure and function, regardless of the amount of collagen. Certainly, fragmentation of collagen and disconnection from the network structure would impair normal aortic function (14).

Smooth muscle cell rarefaction and increased amounts of vacuolated basophilic material and calcification of the smooth muscle cell are evident in TAA (6, 8). These changes not only are to some extent unique but also are an accentuation of the changes in the aorta observed in the normal aging process (15). A minority opinion contends, there is a relative increase in the number of smooth muscle cells after adjusting for the increased surface area of the media in the dilated TAA (16). Regardless, there is a change in smooth muscle cell morphology to a morphology with irregularly shaped cells with distorted intracellular organelles (vacuolated cytoplasm, enlarged endoplasmic reticulum, and decreased amount of myofilaments) and irregularly shaped nuclei (8). Smooth muscle cells in TAA exhibit a well-developed endoplasmic reticulum and Golgi apparatus (6).

The loss of smooth muscle cells in TAA either through change in their functional capacity, i.e., loss of contractile apparatus or through cell death is a feature of TAA (8) that strongly suggests diminution of arterial capacity to contract and maintain its size and shape despite arterial distension from blood pressure.

## Factors Promoting TAA

### Hypertension

**Figure 1:**
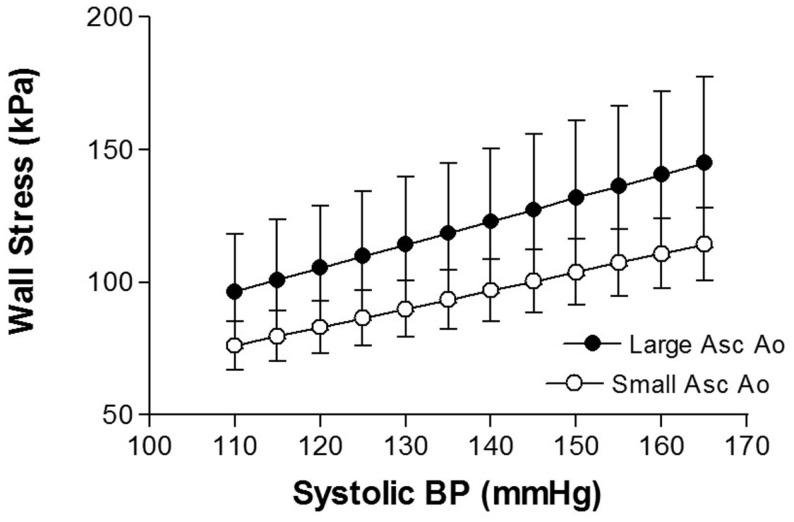
**Shows the relationship between aortic wall stress and systolic blood pressure in thoracic aortic aneurysm of different sizes [from Rabkin and Janusz (21)]**.

Increased blood pressure is associated with TAA (17, 18). Hypertension is also a major factor associated with thoracic aortic dissection (19, 20). The most likely explanation for the link between hypertension and TAA development is the mechanical effects of elevated blood pressure. The increase in stress on the aortic wall resulting from elevations in systolic pressure produces aortic expansion. Using data from patients with ascending aortic aneurysm, we estimated wall stress and found that over a systolic blood pressure range of 110–165 mmHg, there was approximately 4 kPa increase in wall stress for each 5 mmHg increment in systolic blood pressure (21) (Figure 1). Mean circumferential wall stress in TAA, removed at surgery and tested *in vitro*, increases linearly with increased intraluminal pressure and aortic diameter (22). Thus, arterial blood pressure is a major force promoting arterial enlargement. Arterial distending pressure – pulse pressure, and the rate of rise of pulse pressure (dP/dt) also produce stress on the arterial wall.

The role of hypertension in the creation of TAA is not only a balance between the distending action of increased wall stress and the constraining and restoring forces of the arterial wall’s elastic lamellae, collagen, and smooth muscle but also includes the impact of neurohumeral factors, operative in some patients with hypertension. In addition, genetic factors influence the impact of hypertension on TAA development. A case control study suggested that a variant allele of *THBS2* is a risk factor for TAA in hypertensive patients, whereas the variant alleles of *HSPA8*, *GPX1*, *AGT*, and *TNF* are protective against this condition (23).

Angiotensin II is a potent pressor hormone that plays an important role in human hypertension. Prolonged angiotensin II infusion in experimental animals, mainly the mouse, produces aortic aneurysms primarily not only in the abdominal aorta but also in the thoracic aorta (24–27). Although the mouse model for TAA is of value (28), we must be mindful of simply extrapolating murine data to humans because the structure of the human thoracic aorta is more complex than that of mouse (5).

Antihypertensive treatment reduces aneurysm formation in mice with TAA induced by the combination of agents producing both hypertension and degeneration of the elastic lamina (29). In the absence of randomized clinical studies with TAA as the end point, these experimental studies provide helpful data.

### Age

Aging predisposes the aorta to dilatation through changes in aortic structure and composition. *In vitro* studies of the descending thoracic aortas of persons without known aortic disease show an age-related increase in aortic fragility and susceptibility to permanent dilation of the thoracic aorta after pressure distention (30).

Telomere shortening, a marker of aging, occurs more frequently in patients with TAA compared to controls (31). However, this finding may relate mainly to TAA associated with bicuspid aortic valve and not in non-genetic forms of TAA (32).

## Matrix Metalloproteinases

Matrix metalloproteinases (MMPs) play an important role in connective tissue homeostasis (33). MMPs comprise a large family of Zn^2+^-dependent proteolytic proteases, which are synthesized by a number of the cellular components of the aorta including endothelial cells, smooth muscle cells, fibroblasts, and macrophages (34). Under physiological conditions, the activities of metalloproteinases are precisely regulated at the levels of transcription, zymogen activation, and inhibition by endogenous inhibitors (35–37). Disruption of the balance between the production of active enzymes and their inhibition, favoring MMP activation produces accelerated turnover of extracellular matrix (38). Several MMPs, notably MMP-2 and MMP-9, first identified as gelatinases readily digest collagen and other molecules within the vasculature (33). In addition to its action on extracellular matrix, MMPs also acted on molecules involved in signal transduction (38). Gene expression analysis has found an increase in levels of MMP-2 and MMP-9 in TAA (39). A recent meta-analysis showed that there was a significant increase in MMP-9 in the aorta from persons with TAA compared to persons without TAA (40).

Although the factors producing elastin fiber degradation leading to TAA formation are not completely understood, one proteinase that has elastinolytic activity is matrix metalloproteinase-9, which can be produced by monocytes or macrophage-like cells (41). Matrix metalloproteinase cleavage of elastin display biochemical characterization that suggested that elastin cleavage sites are readily accessible to enzymatic attack (42). Other MMPs likely to be involved in TAA development are MMP-14 and -19 whose expression is increased in TAA (43).

MMP-9 is subject to regulatory control through different signal transduction pathways. AKT2 (RAC-beta serine/threonine-protein kinase) or protein kinase B (PKB) and phospho-AKT levels are significantly reduced in human TAA (44). Aortas from Akt2-deficient mice demonstrate tissue destruction, apoptotic cell death, and inflammatory cell infiltration that were not observed in wild-type mice (44). Angiotensin II-infused Akt2-deficient mice show increased expression of MMP-9 (44).

### Inflammation

A role for inflammation in the pathogenesis of TAA is intriguing especially as the type of TAA being discussed has been labeled as “degenerative.” Leukocyte infiltration is greater in the media than in the intima or adventitia of TAA compared to non-aneurysmal aortas (45). Only 50% of TAA had leukocyte infiltration suggesting that it is certainly not a universal feature of TAA (45). Aneurysms with leukocyte infiltration, however, are significantly larger than the aneurysms without leukocyte infiltration (45).

The media and adventitia of TAA have an increased numbers of T lymphocytes and macrophages when compared with control aortas (46). TAA with transmural inflammation, is distinguished by Th1-type immune responses with activated CD4^+^ and CD8^+^ T lymphocytes which produce IFN-γ (45). The density of helper T cells (CD4^+^) is several-fold higher than cytotoxic T cells in the adventitia of infiltrated aneurysms (45, 47). CD4 and CIITA (Class II transactivator), a major regulator of MHC II transcription, show strikingly higher expression in TAA (48). There are significantly more CD3^+^ cells and CD68^+^ cells in the aortas of patients with TAA than in control aortas (47, 49). CD3^+^ cells are localized in the media and surrounding the vasa vasorum in the adventitia (49). Further supporting the contention of an inflammatory process in TAA is the finding that CRP, a traditional marker of inflammation, correlates with the size (diameter) of both ascending and descending TAAs (50). Indirect evidence linking inflammation and TAA development is the finding that TAAs with evidence of inflammation have less collagen, less elastin, and more elastin fragmentation (45).

**Figure 2:**
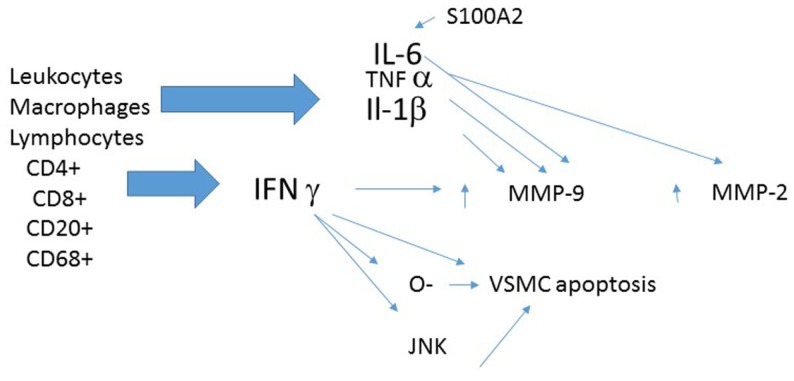
**Shows some of the inflammatory factors that may be operative in production of TAA**.

Several inflammatory mediators are worthy of further discussion (Figure 2).

#### Interferon-gamma

Interferon-gamma (IFN-γ) expression is increased in ascending thoracic aortic aneurysm (ATAA) (45, 47). This finding is consistent with the significant elevations in circulating interferon-γ, interferon-inducible protein-10, interferon-inducible T cell alpha chemoattractant (I-TAC), and monokine induced by IFN-γ in patients with TAA (51). TAA aortas show more IFN-γ-inducible chemokines IP-10 and Mig, and recruitment of lymphocytes bearing their cognate receptor CXCR3 (45). Increased IFN-γ expression in TAA is consistent with the finding that IFN-γ is one of the most consistently up regulated cytokines in large AAAs (52).

Tissue IFN-γ expression correlates with the amount of MMP-9 and the amount of apoptosis in media of TAA (47). IFN-γ may use a JNK signaling pathway to produce MMP-9 activation, apoptosis, and aneurysm formation (47). Additional putative mechanisms for the adverse cellular effect of IFN-γ include enzyme induction and the formation and release of reactive oxygen species (ROS) (53).

Patients with TAA have increased plasma levels of IFN-γ as well as chemokines induced by IFN-γ specifically interferon-inducible protein-10 (IP-10), I-TAC, and monokine induced by interferon-gamma (Mig) IP-10 (51). The degree of elevation, however, does not correlate with either TAA size or its rate of growth (51).

Some TAAs are characterized by recruitment of CXCR3^+^ T cells, in association with secretion of the IFN-γ-inducible chemokines, IP-10, and Mig (45). CXCR-3 the cognate receptor for Ip10, permits recruitment or retention of CD45 leukocytes into the vessel wall and initiates an inflammatory response and subsequent aneurysm formation (51).

Another maker of inflammation, IgG4 was present in the ascending aortic wall of 13% of TAAs and was associated with larger TAA and those more likely to have a dissection (54). While this may represent a specific kind of TAA, it nevertheless assists in linking inflammation to TAA and its consequences.

The anti-inflammatory action of azelnidipine is one putative explanation for its ability to prevents aneurysm progression in angiotensin II and beta-aminopropionitrile treated mice (55). The antihypertensive agent amlodipine when administered to mice in which TAA, is induced by this method, show a lack of adventitial inflammation and medial degeneration (29).

Interleukin-1beta (IL-1β), a pro-inflammatory cytokine, is markedly increased (almost 20-fold) in human TAAs (47, 56). IL-1β can enhance release of elastases (57), which can degrade the elastin component of the arterial wall. Genetic deletion of IL-1β and IL-1R significantly decreased thoracic aortic dilation in an experimental model of TAA (56). Importantly, aortas from IL-1β knockout and IL-1R knockout animals demonstrate preserved elastin and smooth muscle cells, fewer inflammatory cells, and decreased inflammatory cytokine and MMP-9 expression (56). Treatment with the IL-1R antagonist, anakinra, attenuateds TAA development in an experimental model (56). Analogous supporting data is the observation that mice deficient in a negative regulator of IL-1β signaling, interleukin-1 receptor antagonist IL-1Ra-deficient (*IL- 1Ra*-/-) mice, develop femoral artery aneurysms with histologic evidence of elastin degradation (58).

#### TNF-α

TNF-α reduces aortic elastin as TNF-α, as well as basic fibroblast growth factor, reduces elastin gene transcription in aortic smooth muscle cells (57). TNF-α also promotes elastin breakdown through enhanced release of MMP-2 and MMP-9 by vascular smooth muscle cells (57).

#### S100A12

S100A12, a pro-inflammatory protein that activates the receptor for advanced glycation end products (RAGE), is increased in 25% of TAA (59). Its presence is associated with an increased risk of dissection (59). Transgenic mice, overexpressing S100A12 show disarray of elastic fibers, and increased collagen deposition in the aortic wall as well as aortic dilatation (60). These mice also show an increase in MMP-2 protein and reduction in smooth muscle stress fibers (60).

#### IL-6

IL-6 is also increased in TAA (50). Further, there is a significant correlation between IL-6 and CRP and the size (diameter) of both [both ascending and descending aneurysms (50)]. IL-6 can be turned on by S100A12 (60). IL-1β as well as IFN-γ might affect the formation of TAA through the up-regulation of MMP-9 and the apoptosis cells in human aortic media (47).

#### Transforming Growth Factor-Beta

Transforming growth factor-beta (TGF-β) has been implicated in certain genetic causes of TAA and TGF-β and/or its signaling pathways can be abnormal in the arterial walls of degenerative TAA; however, the precise role and molecular mechanisms by which TGF-β might be operative in TAA has been characterized as elusive and controversial (61). Indeed, investigators have implicated both enhanced TGF-β signaling and loss of TGF-β function (TGF-β receptor mutations) in aneurysm formation (62, 63). A more detailed discussion of this subject is available in several reviews (61, 62, 64). One speculation, which might unify the disparate results, is that each cell type within the aortic wall responds differently to TGF-β and it is the balance of effects of TGF-β in any situation, as well as the impact of other factors, which dictate the net effect (62). TGF-β has the potential to produce smooth muscle cell apoptosis and stimulate the differentiation of fibroblasts into myofibroblasts, which can aid in TAA formation but it also can down regulate the activity of MMPs, which might reduce TAA development. From the perspective of this review whose objective is presenting accepted promoters and reducers of TAA development, TGF-β will not be considered further.

#### A Disintegrin and Metalloproteinase with Thrombospondin Motifs

A disintegrin and metalloproteinase with thrombospondin motifs (ADAMTS) may promote TAA development by degrading versican and facilitating macrophage invasion into the aortic wall. ADAMTS-1 and ADAMTS-4 protein and mRNA expression are increased in TAA (39, 65). ADAMTS-1 and ADAMTS-4 were identified in vascular smooth muscle cells and macrophages in TAA concomitant with degradation of versican the main proteoglycan substrate of ADAMTS proteinases in the aorta (65).

## Coagulation/Fibrinolytic Systems

### Plasmin

Plasmin is generated from the zymogen plasminogen by tissue-type plasminogen activator (t-PA) and urokinase-type plasminogen activator (u-PA). Plasmin has been implicated as a causative factor for TAA development based on several lines of evidence. First, free t-PA is present in increased amounts in aneurysmal thoracic aortic wall compared to normal aorta (6). Second, Apo E deficient mice that are also deficient in t-PA (Apoe^−/−^:Plat^−/−^) or u-PA (Apo e^−/−^:Plau^−/−^) do not have vascular aneurysms, which occur in Apo E deficient mice that are not deficient in t-PA or u-PA (66). Third, large amounts of t-PA are associated with enlarged arteries (66). Fourth, there is overexpression of the plasminogen activators t-PA and u-PA in TAA (67). These data suggest that plasmin generation is a causative factor for TAA development. One of the sources of t-PA in TAA is macrophages, which invade the vessel wall from the luminal surface and migrate to the media (66). Another source of t-PA and u-PA is the aortic smooth muscle, which is transformed into a secretory cell in TAA (6).

**Figure 3:**
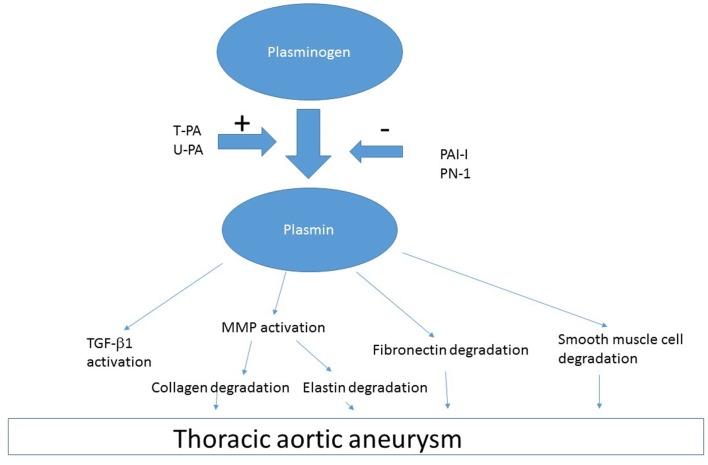
**Shows factors in coagulation cascade that produce or oppose development of TAA**.

Plasminogen and t-PA can bind to external cell surface via annexin II and produce plasmin (68). Left unchecked, excess t-PA and u-PA increases plasmin generation, which can act on various proteins (Figure 3). Plasmin activation of various factors including MMP can lead to degradation of essential constituents of the aortic wall including elastin, collagen, fibronectin, and laminin (69). Plasmin-induced activation of MMP-3, -9, -12, and -13 produces collagen and elastin degradation (66). There is likely a multiplier effect from the damage to each of these constituents of the arterial wall, because there are major binding interaction between fibrillins and fibronectin, which involves the collagen/gelatin-binding region between domains FNI(6) and FNI(9) (70). Thus, destruction of one element of vasculature has importance for the entire structure as the cellular elements of the aorta are interconnected.

Plasmin, produced by t-PA or u-PA, can also activate pathways leading to smooth muscle cell apoptosis (71) producing the histologic picture of smooth muscle cell rarefaction (6). Plasmin-induced apoptosis can be independent of MMP (72). Plasmin can also cleave cytokines that are present as zymogens within the arterial wall such as TGF-β (73). Deficiency of u-PA protects against media destruction and aneurysm formation, probably through reduction of plasmin-dependent activation of pro-MMPs (66).

### Platelet and prothrombin activation

Platelets and prothrombin are activated in patients with TAA >45 mm in diameter (74). Circulating markers of platelet activation (sGPV, sCD40L) are significantly higher in plasma of patients with TAA (74). The absence of a difference in these factors between Marfan’s or degenerative TAA, suggests that platelet activation is not an etiology of degenerative TAA but rather is either a consequence of the altered hemodynamics (rheology) in TAA or is a later stage factors that may or may not accentuate TAA expansion.

Prothrombin/thrombin (II/IIa) (immunoreativity) is present in the aortic wall of TAA but not in the normal aorta (74). Thrombin can damage the endothelial layer of cells (75, 76). The consequence of endothelial barrier dysfunction (76) can be the entry of circulating factors into the arterial wall, which may damage arterial wall components leading to loss of aortic structure and function.

### SMAD-2

Although SMAD-2 is more well known for its presence in specific genetic causes of TAA, there are data that dysregulation of SMAD2 activation and its nuclear translocation occur in degenerative TAA as well (77). SMAD2 protein and phosphorylated SMAD2 are increased in the medial layer of TAA of a degenerative cause (78). In contrast, SMAD3 as well as RhoA pathways are not altered in TAA (78). The activation and the overexpression of SMAD2 are specifically found in smooth muscle cells of TAA (78).

To the extent that angiotensin II is operative in arterial hypertension, it is noteworthy that angiotensin II produces SMAD2 activation leading to MMP-9 production through a pathway involving intracellular signal regulated kinase (ERK) (79). In an experimental model of surgically induced TAA in mice (C57BL/6J), protein levels of SMAD2, SMAD1/5/8, and phospho-SMAD1/5/8 were increased and SMAD4 was decreased from control values (80). A switch from a TGF-βR(I)/SMAD2-dependent response, to an ALK-1/SMAD1/5/8 response may enhance matrix degradation leading to TAA development (80).

## Factors Opposing TAA Development

Using a concept of accentuating and mitigating factors, one can visualize the construct leading to TAA formation as an imbalance between factors favoring and factors opposing permanent aortic expansion.

## Arterial Wall Composition

**Figure 4:**
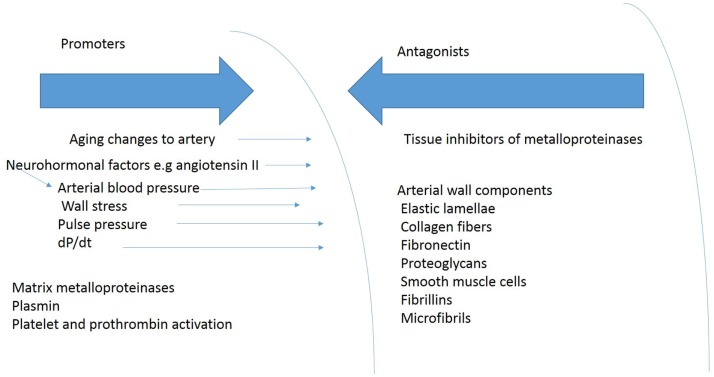
**Shows some of the blood pressure and vascular factors that produce or oppose development of TAA**.

The constituents of the aortic wall maintain normal aortic dimensions in the face of aortic wall stress (Figure 4). Reductions in the amount of any of these components or their fragmentations diminish the ability of the arterial wall to resist permanent deformation. Loss of elastin produces tortuous, stiff vessels that show little diameter change between systole and diastole and become stenotic because of smooth muscle cell proliferation, presumably in an attempt to compensate for the loss of elastin (14). The loss of elastin may increase blood pressure leading to a further increase in circumferential stress, which creates a further impetus for aortic aneurysm expansion. The relative amounts of changes in arterial wall components – collagen, elastin, glycosaminoglycans, fibrulins, fibronectin, and vascular smooth muscle cells – in the aortic segments that experience these changes, may influence aortic compliance in TAA (81).

## Tissue Inhibitors of Metalloproteinases

Tissue inhibitors of metalloproteinases (TIMPs) are endogenous inhibitors of MMPs and consequently are important regulators of local MMP activity (35, 38). The four tissue inhibitor of metalloproteinases (TIMP-1 to -4) are broad-spectrum MMP inhibitors but with some differences in specificity (38, 82). These proteins are significant regulators of the activities of MMPs and, in some instances, of other metalloendopeptidases as well (83). Tissue effects are determined by the balance of MMP and TIMP activation. A recent meta-analysis showed that there was a highly significant reduction in TIMP-1 and TIMP-2 in TAA compared to controls without TAA, resulting in a MMP-9 to TIMP-1 or TIMP-2 ratio over 3.5-fold greater than controls (40).

In Akt2-deficient mice, angiotensin reduces the expression of TIMP-1, which might account for the TAA found in this animal model (44).

## Inhibitors of MMP

### microRNAs

Small non-coding RNA molecules or microRNAs (miRs) can induce translational repression and modulate protein abundance of specific genes products and in so doing affect cellular function generally and cardiovascular tissue specifically (84). Decreases in miRs-1, -21, -29a, -133a, and -486 expression is present in TAA and there is a significant relationship between miR expression levels (miRs-1, -21, -29a, and -133a) and aortic diameter (85). MMP-2 and MMP-9 are potential targets for miR-29a and miR-133a (85). These data plus the significant inverse relationship between miR-29a and total MMP-2 in TAA suggest that there is an inhibitory signal that normally attenuates MMP production so that a reduction in these inhibitors can lead to TAA formation (85).

In ATAAs with dissection seven miRNAs were markedly different from normal aorta (86). Target gene-related pathway analysis point to five pathways, especially those involved in the focal adhesion and the mitogen-activated protein kinase (MAPK) signaling pathways (86). mir143/145 under expression that may explain part of the changes in vascular smooth muscle in TAA (86).

## Inhibitors of Plasmin Production

Plasminogen activator inhibitor-1 (PAI-1) mRNA and protein are increased in the media of TAA (67). In vascular smooth muscle cells isolated from human TAA, there is an increased amount of PAI-1, which increases further when exposed to TGF-β 1 (67). PAI-1 is subject to regulatory control by the SMAD2 signaling pathway (67). PAI protects against plasmin-induced vascular smooth muscle cell death (67). PAI-1 should protect against TAA development based on the role for plasmin in TAA formation discussed above. There is little data in this area; however, studies in AAA support the concept of PAI-I as an inhibitor of aneurysm formation (87, 88). PAI-I can alter aortic diameter in experimentally induced AAA (89).

Protease nexin-1 (PN-1), a regulator of protease activities in the vascular wall, is an inhibitor of thrombin, urokinase-type plasminogen activator (u-PA), tissue-type plasminogen activator (t-PA), and plasmin (90). PN-1 is produced and secreted by VSMCs (91) and is present in large amounts on cell membranes (90). PN-1 mRNA and protein is overexpressed in the media of TAA and in vascular smooth muscle cells cultured from TAA (67). PN-1 overexpression promotes development of an anti-proteolytic vascular smooth muscle cell phenotype (67). PN-1 overexpression inhibits plasmin and tissue-type plasminogen activator via the formation of inhibitory complexes and prevents cell apoptosis (69).

The increase expression of PAI-I and PN-1 in TAA suggested that these factors are part of cell defense mechanisms against protease-induced matrix degradation and protease-induced cell death (90).

## Syndecans

Syndecans are a family of four cell surface proteoglycans [syndecan-1 (Sdc-1), Sdc-2, Sdc-3, and Sdc-4] that interacts with a number of soluble and insoluble factors in the extracellular matrix to regulate transmembrane signaling events (92). Syndecans have diverse cellular roles including regulation of extracellular matrix assembly, tissue repair, as well as inflammation (93).

Although there are no data on Syndecans in TAA, it is noteworthy that Sdc-1 deficiency exacerbated AAA formation in experimental AAA and is associated with protease activity, elastin degradation, and inflammatory cell recruitment into the aortic wall (94). Tumor-associated MMPs cleave the ectodomains of human syndecan-1 and syndecan-4 (95). Although the process has not been studied in TAA, it is interesting to speculate that MMP activation in TAA may blunt the anti-inflammatory protective actions of Syndecans in TAA. Syndecan-1 increases the uptake of PN-1 (96), which inhibits the effects of coagulation factors in TAA development.

## Summary

In summary, the processes leading to the formation of TAA are complex, interwoven, and involve promoters and inhibitors at many potential sites. There is a need for more investigation into modulating the initiators and enhancing the inhibitors of TAA.

## Conflict of Interest Statement

The author declares that the research was conducted in the absence of any commercial or financial relationships that could be construed as a potential conflict of interest.

## References

[1] Coady MA Rizzo JA Goldstein LJ Elefteriades JA Natural history, pathogenesis, and etiology of thoracic aortic aneurysms and dissections. Cardiol Clin (1999) 17:615–35. 10.1016/S0733-8651(05)70105-3 10589336

[2] Olsson C Thelin S Stahle E Ekbom A Granath F. Thoracic aortic aneurysm and dissection: increasing prevalence and improved outcomes reported in a nationwide population- based study of more than 14,000 cases from 1987 to 2002. Circulation (2006) 114:2611–8. 10.1161/CIRCULATIONAHA.106.630400 17145990

[3] Norman PE Powell JT Site specificity of aneurysmal disease. Circulation (2010) 121:560–8. 10.1161/CIRCULATIONAHA.109.880724 20124136

[4] Wolinsky H Glagov S A lamellar unit of aortic medial structure and function in mammals. Circ Res (1967) 20:99–111. 10.1161/01.RES.20.1.99 4959753

[5] Dingemans KP Teeling P Lagendijk JH Becker AE. Extracellular matrix of the human aortic media: an ultrastructural histochemical and immunohistochemical study of the adult aortic media. Anat Rec (2000) 258:1–14. 10.1002/(SICI)1097-0185(20000101)258:1<1::AID-AR1>3.0.CO;2-7 10603443

[6] Borges LF Gomez D Quintana M Touat Z Jondeau G Leclercq A Fibrinolytic activity is associated with presence of cystic medial degeneration in aneurysms of the ascending aorta. Histopathology (2010) 57:917–32. 10.1111/j.1365-2559.2010.03719.x 21166705

[7] Lindeman JH Ashcroft BA Beenakker JW van Es M Koekkoek NB Prins FA Distinct defects in collagen microarchitecture underlie vessel-wall failure in advanced abdominal aneurysms and aneurysms in Marfan syndrome. Proc Natl Acad Sci U S A (2010) 107:862–5. 10.1073/pnas.0910312107 20080766 PMC2818895

[8] Lesauskaite V Tanganelli P Sassi C Neri E Diciolla F Ivanoviene L Smooth muscle cells of the media in the dilatative pathology of ascending thoracic aorta: morphology, immunoreactivity for osteopontin, matrix metalloproteinases, and their inhibitors. Hum Pathol (2001) 32:1003–11. 10.1053/hupa.2001.27107 11567232

[9] Humphrey JD Possible mechanical roles of glycosaminoglycans in thoracic aortic dissection and associations with dysregulated transforming growth factor-beta. J Vasc Res (2013) 50:1–10. 10.1159/000342436 23018968 PMC3607504

[10] Matsumoto K Maniwa T Tanaka T Satoh K Okunishi H Oda T. Proteomic analysis of calcified abdominal and thoracic aortic aneurysms. Int J Mol Med (2012) 30:417–29. 10.3892/ijmm.2012.985 22552374

[11] Wang X LeMaire SA Chen L Carter SA Shen YH Gan Y Decreased expression of fibulin-5 correlates with reduced elastin in thoracic aortic dissection. Surgery (2005) 138:352–9. 10.1016/j.surg.2005.06.006 16153447

[12] Meng YH Tian C Liu L Wang L Chang Q. Elevated expression of connective tissue growth factor, osteopontin and increased collagen content in human ascending thoracic aortic aneurysms. Vascular (2014) 22:20–7. 10.1177/1708538112472282 23508392

[13] Toumpoulis IK Oxford JT Cowan DB Anagnostopoulos CE Rokkas CK Chamogeorgakis TP Differential expression of collagen type V and XI alpha-1 in human ascending thoracic aortic aneurysms. Ann Thorac Surg (2009) 88:506–13. 10.1016/j.athoracsur.2009.04.030 19632402 PMC2834780

[14] Wagenseil JE Mecham RP. Vascular extracellular matrix and arterial mechanics. Physiol Rev (2009) 89:957–89. 10.1152/physrev.00041.2008 19584318 PMC2775470

[15] Schlatmann TJ Becker AE. Pathogenesis of dissecting aneurysm of aorta. Comparative histopathologic study of significance of medial changes. Am J Cardiol (1977) 39:21–6. 10.1016/S0002-9149(77)80005-2 831424

[16] Tang PC Coady MA Lovoulos C Dardik A Aslan M Elefteriades JA Hyperplastic cellular remodeling of the media in ascending thoracic aortic aneurysms. Circulation (2005) 112:1098–105. 10.1161/CIRCULATIONAHA.104.511717 16116068

[17] Ince H Nienaber CA. Etiology, pathogenesis and management of thoracic aortic aneurysm. Nat Clin Pract Cardiovasc Med (2007) 4:418–27. 10.1038/ncpcardio0937 17653114

[18] LeMaire SA Russell L Epidemiology of thoracic aortic dissection. Nat Rev Cardiol (2011) 8:103–13. 10.1038/nrcardio.2010.187 21173794

[19] Pape LA Tsai TT Isselbacher EM Oh JK O’Gara PT Evangelista A Aortic diameter >=5.5 cm is not a good predictor of type A aortic dissection: observations from the international registry of acute aortic dissection (IRAD). Circulation (2007) 116:1120–7. 10.1161/CIRCULATIONAHA.107.702720 17709637

[20] Chan KK Rabkin SW. Increasing prevalence of hypertension among patients with thoracic aorta dissection: trends over eight decades – a structured meta-analysis. Am J Hypertens (2014) 27(7):907–17. 10.1093/ajh/hpt293 24522500

[21] Rabkin S Janusz M. Aortic wall stress in hypertension and ascending thoracic aortic aneurysms: implications for antihypertensive therapy. High Blood Press Cardiovasc Prev (2013) 20:265–71. 10.1007/s40292-013-0026-z 24092647

[22] Okamoto RJ Wagenseil JE DeLong WR Peterson SJ Kouchoukos NT Sundt TM III. Mechanical properties of dilated human ascending aorta. Ann Biomed Eng (2002) 30:624–35. 10.1114/1.1484220 12108837

[23] Kato K Oguri M Kato N Hibino T Yajima K Yoshida T Assessment of genetic risk factors for thoracic aortic aneurysm in hypertensive patients. Am J Hypertens (2008) 21:1023–7. 10.1038/ajh.2008.229 18600213

[24] Moltzer E Essers J van Esch JH Roos-Hesselink JW Danser AH. The role of the renin-angiotensin system in thoracic aortic aneurysms: clinical implications. Pharmacol Ther (2011) 131:50–60. 10.1016/j.pharmthera.2011.04.002 21504760

[25] Lu H Rateri DL Bruemmer D Cassis LA Daugherty A. Involvement of the renin- angiotensin system in abdominal and thoracic aortic aneurysms. Clin Sci (2012) 123:531–43. 10.1042/CS20120097 22788237

[26] Owens AP III Subramanian V Moorleghen JJ Guo Z McNamara CA Cassis LA Angiotensin II induces a region-specific hyperplasia of the ascending aorta through regulation of inhibitor of differentiation 3. Circ Res (2010) 106:611–9. 10.1161/CIRCRESAHA.109.212837 20019328 PMC2825288

[27] Daugherty A Rateri DL Charo IF Owens AP Howatt DA Cassis LA. Angiotensin II infusion promotes ascending aortic aneurysms: attenuation by CCR2 deficiency in apoE-/- mice. Clin Sci (2010) 118:681–9. 10.1042/CS20090372 20088827 PMC2841499

[28] Bruemmer D Daugherty A Lu H Rateri DL. Relevance of angiotensin II-induced aortic pathologies in mice to human aortic aneurysms. Ann N Y Acad Sci (2011) 1245:7–10. 10.1111/j.1749-6632.2011.06332.x 22211965

[29] Kanematsu Y Kanematsu M Kurihara C Tsou TL Nuki Y Liang EI Pharmacologically induced thoracic and abdominal aortic aneurysms in mice. Hypertension (2010) 55:1267–74. 10.1161/HYPERTENSIONAHA.109.140558 20212272 PMC2859958

[30] Groenink M Langerak SE Vanbavel E van der Wall EE Mulder BJ van der Wal AC The influence of aging and aortic stiffness on permanent dilation and breaking stress of the thoracic descending aorta. Cardiovasc Res (1999) 43:471–80. 10.1016/S0008-6363(99)00095-4 10536677

[31] Balistreri CR Pisano C Merlo D Fattouch K Caruso M Incalcaterra E Is the mean blood leukocyte telomere length a predictor for sporadic thoracic aortic aneurysm? Data from a preliminary study. Rejuvenation Res (2012) 15:170–3. 10.1089/rej.2011.1273 22533425

[32] Blunder S Messner B Aschacher T Zeller I Turkcan A Wiedemann D Characteristics of TAV- and BAV-associated thoracic aortic aneurysms – smooth muscle cell biology, expression profiling, and histological analyses. Atherosclerosis (2012) 220:355–61. 10.1016/j.atherosclerosis.2011.11.035 22178424

[33] Nagase H Visse R Murphy G. Structure and function of matrix metalloproteinases and TIMPs. Cardiovasc Res (2006) 69:562–73. 10.1016/j.cardiores.2005.12.002 16405877

[34] Galis ZS Khatri JJ. Matrix metalloproteinases in vascular remodeling and atherogenesis: the good, the bad, and the ugly. Circ Res (2002) 90(3):251–62. 10.1161/hh0302.105345 11861412

[35] Bode W Fernandez-Catalan C Tschesche H Grams F Nagase H Maskos K Structural properties of matrix metalloproteinases. Cell Mol Life Sci (1999) 55:639–52. 10.1007/s000180050320 10357232 PMC11146962

[36] Clark IM Swingler TE Sampieri CL Edwards DR. The regulation of matrix metalloproteinases and their inhibitors. Int J Biochem Cell Biol (2008) 40:1362–78. 10.1016/j.biocel.2007.12.006 18258475

[37] Vandooren J Van den Steen PE Opdenakker G. Biochemistry and molecular biology of gelatinase B or matrix metalloproteinase-9 (MMP-9): the next decade. Crit Rev Biochem Mol Biol (2013) 48:222–72. 10.3109/10409238.2013.770819 23547785

[38] Brew K Nagase H. The tissue inhibitors of metalloproteinases (TIMPs): an ancient family with structural and functional diversity. Biochim Biophys Acta (2010) 1803:55–71. 10.1016/j.bbamcr.2010.01.003 20080133 PMC2853873

[39] Taketani T Imai Y Morota T Maemura K Morita H Hayashi D Altered patterns of gene expression specific to thoracic aortic aneurysms: microarray analysis of surgically resected specimens. Int Heart J (2005) 46:265–77. 10.1536/ihj.46.265 15876810

[40] Rabkin SW. Differential expression of MMP-2, MMP-9 and TIMP proteins in ascending thoracic aortic aneurysm – comparison with and without bicuspid aortic valve: a meta-analysis. Vasa (2014) 43(6):433–42. 10.1024/0301-1526/a000390 25339161

[41] Katsuda S Okada Y Imai K Nakanishi I. Matrix metalloproteinase-9 (92-kd gelatinase/type IV collagenase equals gelatinase B) can degrade arterial elastin. Am J Pathol (1994) 145:1208–18. 7977651 PMC1887414

[42] Mecham RP Broekelmann TJ Fliszar CJ Shapiro SD Welgus HG Senior RM. Elastin degradation by matrix metalloproteinases. Cleavage site specificity and mechanisms of elastolysis. J Biol Chem (1997) 272:18071–6. 10.1074/jbc.272.29.18071 9218437

[43] Jackson V Olsson T Kurtovic S Folkersen L Paloschi V Wagsater D Matrix metalloproteinase 14 and 19 expression is associated with thoracic aortic aneurysms. J Thorac Cardiovasc Surg (2012) 144:459–66. 10.1016/j.jtcvs.2011.08.043 21955474

[44] Shen YH Zhang L Ren P Nguyen MT Zou S Wu D AKT2 confers protection against aortic aneurysms and dissections. Circ Res (2013) 112:618–32. 10.1161/CIRCRESAHA.112.300735 23250987 PMC3586338

[45] Tang PC Yakimov AO Teesdale MA Coady MA Dardik A Elefteriades JA Transmural inflammation by interferon-gamma-producing T cells correlates with outward vascular remodeling and intimal expansion of ascending thoracic aortic aneurysms. FASEB J (2005) 19(11):1528–30. 10.1096/fj.05-3671fje 16014397

[46] He R Guo DC Sun W Papke CL Duraisamy S Estrera AL Characterization of the inflammatory cells in ascending thoracic aortic aneurysms in patients with Marfan syndrome, familial thoracic aortic aneurysms, and sporadic aneurysms. J Thorac Cardiovasc Surg (2008) 136:922–929 929.e1. 10.1016/j.jtcvs.2007.12.063 18954631 PMC2590650

[47] Zhang L Liao MF Tian L Zou SL Lu QS Bao JM Overexpression of interleukin-1beta and interferon- in type I thoracic aortic dissections and ascending thoracic aortic aneurysms: possible correlation with matrix metalloproteinase-9 expression and apoptosis of aortic media cells. Eur J Cardiothorac Surg (2011) 40:17–22. 10.1016/j.ejcts.2010.09.019 21349736

[48] Folkersen L Wagsater D Paloschi V Jackson V Petrini J Kurtovic S Unraveling divergent gene expression profiles in bicuspid and tricuspid aortic valve patients with thoracic aortic dilatation: the ASAP study. Mol Med (2011) 17:1365–73. 10.2119/molmed.2011.00286 21968790 PMC3321821

[49] He R Guo DC Estrera AL Safi HJ Huynh TT Yin Z Characterization of the inflammatory and apoptotic cells in the aortas of patients with ascending thoracic aortic aneurysms and dissections. J Thorac Cardiovasc Surg (2006) 131:671–8. 10.1016/j.jtcvs.2005.09.018 16515922

[50] Artemiou P Charokopos N Rouska E Sabol F Chrysogonidis I Tsavdaridou V C-reactive protein/interleukin-6 ratio as marker of the size of the uncomplicated thoracic aortic aneurysms. Interact Cardiovasc Thorac Surg (2012) 15:871–7. 10.1093/icvts/ivs331 22843654 PMC3480603

[51] Gallo A Saad A Ali R Dardik A Tellides G Geirsson A. Circulating interferon – inducible Cys-X-Cys chemokine receptor 3 ligands are elevated in humans with aortic aneurysms and Cys-X-Cys chemokine receptor 3 is necessary for aneurysm formation in mice. J Thorac Cardiovasc Surg (2012) 143:704–10. 10.1016/j.jtcvs.2011.08.036 21962843 PMC4526250

[52] Golledge AL Walker P Norman PE Golledge J. A systematic review of studies examining inflammation associated cytokines in human abdominal aortic aneurysm samples. Dis Markers (2009) 26:181–8. 10.3233/DMA-2009-0629 19729799 PMC3833704

[53] Schroecksnadel K Frick B Winkler C Fuchs D. Crucial role of interferon-gamma and stimulated macrophages in cardiovascular disease. Curr Vasc Pharmacol (2006) 4:205–13. 10.2174/157016106777698379 16842138

[54] Kajander H Paavonen T Valo T Tarkka M Mennander AA. Immunoglobulin G4- positive ascending thoracic aortitis may be prone to dissection. J Thorac Cardiovasc Surg (2013) 146:1449–55. 10.1016/j.jtcvs.2012.09.039 23062412

[55] Hirata Y Kurobe H Akaike M Chikugo F Hori T Bando Y Enhanced inflammation in epicardial fat in patients with coronary artery disease. Int Heart J (2011) 52:139–42. 10.1536/ihj.52.139 21646734

[56] Johnston WF Salmon M Pope NH Meher A Su G Stone ML Inhibition of interleukin-1beta decreases aneurysm formation and progression in a novel model of thoracic aortic aneurysms. Circulation (2014) 130:S51–9. 10.1161/CIRCULATIONAHA.113.006800 25200056 PMC5097450

[57] Sproul EP Argraves WS. A cytokine axis regulates elastin formation and degradation. Matrix Biol (2013) 32:86–94. 10.1016/j.matbio.2012.11.004 23160093 PMC3633528

[58] Isoda K Kitagaki M Niida T Kondo H Matsubara O Kikuchi M Deficiency of interleukin-1 receptor antagonist promotes spontaneous femoral artery aneurysm formation in mice. Am J Pathol (2012) 180:1254–63. 10.1016/j.ajpath.2011.11.028 22245214

[59] Das D Gawdzik J Dellefave-Castillo L McNally EM Husain A Raman J S100A12 expression in thoracic aortic aneurysm is associated with increased risk of dissection and perioperative complications. J Am Coll Cardiol (2012) 60:775–85. 10.1016/j.jacc.2012.04.027 22818064 PMC3422448

[60] Hofmann Bowman M Wilk J Heydemann A Kim G Rehman J Lodato JA S100A12 mediates aortic wall remodeling and aortic aneurysm. Circ Res (2010) 106:145–54. 10.1161/CIRCRESAHA.109.209486 19875725 PMC2878187

[61] Gillis E Van Laer L Loeys BL. Genetics of thoracic aortic aneurysm: at the crossroad of transforming growth factor-beta signaling and vascular smooth muscle cell contractility. Circ Res (2013) 113:327–40. 10.1161/CIRCRESAHA.113.300675 23868829

[62] Jones JA Spinale FG Ikonomidis JS. Transforming growth factor-beta signaling in thoracic aortic aneurysm development: a paradox in pathogenesis. J Vasc Res (2009) 46:119–37. 10.1159/000151766 18765947 PMC2645475

[63] Frutkin AD Otsuka G Stempien-Otero A Sesti C Du L Jaffe M TGF-[beta]1 limits plaque growth, stabilizes plaque structure, and prevents aortic dilation in apolipoprotein E-null mice. Arterioscler Thromb Vasc Biol (2009) 29:1251–7. 10.1161/ATVBAHA.109.186593 19325140 PMC2740721

[64] Lin F Yang X. TGF-beta signaling in aortic aneurysm: another round of controversy. J Genet Genomics (2010) 37:583–91. 10.1016/S1673-8527(09)60078-3 20933212

[65] Ren P Zhang L Xu G Palmero LC Albini PT Coselli JS ADAMTS-1 and ADAMTS-4 levels are elevated in thoracic aortic aneurysms and dissections. Ann Thorac Surg (2013) 95:570–7. 10.1016/j.athoracsur.2012.10.084 23245439 PMC3593585

[66] Carmeliet P Moons L Lijnen R Baes M Lemaitre V Tipping P Urokinase-generated plasmin activates matrix metalloproteinases during aneurysm formation. Nat Genet (1997) 17:439–44. 10.1038/ng1297-439 9398846

[67] Gomez D Kessler K Borges LF Richard B Touat Z Ollivier V SMAD2-dependent protease nexin-1 overexpression differentiates chronic aneurysms from acute dissections of human ascending aorta. Arterioscler Thromb Vasc Biol (2013) 33:2222–32. 10.1161/ATVBAHA.113.301327 23814118

[68] Hajjar KA Jacovina AT Chacko J. An endothelial cell receptor for plasminogen/tissue plasminogen activator. I. Identity with annexin II. J Biol Chem (1994) 269:21191–7. 8063740

[69] Rossignol P Ho-Tin-Noe B Vranckx R Bouton MC Meilhac O Lijnen HR Protease nexin-1 inhibits plasminogen activation-induced apoptosis of adherent cells. J Biol Chem (2004) 279:10346–56. 10.1074/jbc.M310964200 14699093

[70] Sabatier L Chen D Fagotto-Kaufmann C Hubmacher D McKee MD Annis DS Fibrillin assembly requires fibronectin. Mol Biol Cell (2009) 20:846–58. 10.1091/mbc.E08-08-0830 19037100 PMC2633374

[71] Meilhac O Ho-Tin-Noe B Houard X Philippe M Michel JB Angles-Cano E. Pericellular plasmin induces smooth muscle cell anoikis. FASEB J (2003) 17(10):1301–3. 10.1096/fj.02-0687fje 12738809

[72] Martin-Ventura JL Madrigal-Matute J Martinez-Pinna R Ramos-Mozo P Blanco-Colio LM Moreno JA Erythrocytes, leukocytes and platelets as a source of oxidative stress in chronic vascular diseases: detoxifying mechanisms and potential therapeutic options. Thromb Haemost (2012) 108:435–42. 10.1160/TH12-04-0248 22836558

[73] Jenkins G. The role of proteases in transforming growth factor-beta activation. Int J Biochem Cell Biol (2008) 40:1068–78. 10.1016/j.biocel.2007.11.026 18243766

[74] Touat Z Lepage L Ollivier V Nataf P Hvass U Labreuche J Dilation-dependent activation of platelets and prothrombin in human thoracic ascending aortic aneurysm. Arterioscler Thromb Vasc Biol (2008) 28:940–6. 10.1161/ATVBAHA.107.158576 18292393

[75] Lum H Malik AB. Regulation of vascular endothelial barrier function. Am J Physiol (1994) 267:223–41. 7943249 10.1152/ajplung.1994.267.3.L223

[76] Birukova AA Birukov KG Smurova K Adyshev D Kaibuchi K Alieva I Novel role of microtubules in thrombin-induced endothelial barrier dysfunction. FASEB J (2004) 18:1879–90. 10.1096/fj.04-2328com 15576491

[77] Gomez D Al Haj Zen A Borges LF Philippe M Gutierrez PS Jondeau G Syndromic and non-syndromic aneurysms of the human ascending aorta share activation of the SMAD2 pathway. J Pathol (2009) 218:131–42. 10.1002/path.2516 19224541

[78] Gomez D Coyet A Ollivier V Jeunemaitre X Jondeau G Michel JB Epigenetic control of vascular smooth muscle cells in Marfan and non-Marfan thoracic aortic aneurysms. Cardiovasc Res (2011) 89:446–56. 10.1093/cvr/cvq291 20829218 PMC3020128

[79] Nagasawa A Yoshimura K Suzuki R Mikamo A Yamashita O Ikeda Y Important role of the angiotensin II pathway in producing matrix metalloproteinase-9 in human thoracic aortic aneurysms. J Surg Res (2013) 183:472–7. 10.1016/j.jss.2012.12.012 23295196

[80] Jones JA Barbour JR Stroud RE Bouges S Stephens SL Spinale FG Altered transforming growth factor-beta signaling in a murine model of thoracic aortic aneurysm. J Vasc Res (2008) 45:457–68. 10.1159/000127437 18434745 PMC2574785

[81] Rabkin S Chan KK Chow B Janusz M. Pulse wave velocity involving proximal portions of the aorta correlates with the degree of aortic dilatation at the sinuses of valsalva in asscending thoracic aortic aneuryems. Ann Vasc Dis (2014) 7(4):404–9. 10.3400/avd.oa.14-00063 25593626 PMC4293191

[82] Khokha R Murthy A Weiss A. Metalloproteinases and their natural inhibitors in inflammation and immunity. Nat Rev Immunol (2013) 13:649–65. 10.1038/nri3499 23969736

[83] Murphy G. Tissue inhibitors of metalloproteinases. Genome Biol (2011) 12:233. 10.1186/gb-2011-12-11-233 22078297 PMC3334591

[84] Small E Frost R Olson E microRNAs add a new dimension to cardiovascular disease. Circulation (2010) 121:1022–32. 10.1161/CIRCULATIONAHA.109.889048 20194875 PMC2847432

[85] Jones JA Stroud RE O’Quinn EC Black LE Barth JL Elefteriades JA Selective microRNA suppression in human thoracic aneurysms: relationship of miR-29a to aortic size and proteolytic induction. Circ Cardiovasc Genet (2011) 4:605–13. 10.1161/CIRCGENETICS.111.960419 22010139 PMC3246193

[86] Liao M Zou S Weng J Hou L Yang L Zhao Z A microRNA profile comparison between thoracic aortic dissection and normal thoracic aorta indicates the potential role of microRNAs in contributing to thoracic aortic dissection pathogenesis. J Vasc Surg (2011) 53:1341.e–1349.e. 10.1016/j.jvs.2010.11.113 21334170

[87] Krikova V Korabecna M Kocova J Treska V Molacek J Tonar Z Quantification of plasminogen activator inhibitor type 1 in the aortic wall. Int Angiol (2009) 28(1):44–9. 19190555

[88] Defawe OD Colige A Lambert CA Munaut C Delvenne P Lapiere CM TIMP-2 and PAI-1 mRNA levels are lower in aneurysmal as compared to athero-occlusive abdominal aortas. Cardiovasc Res (2003) 60:205–13. 10.1016/S0008-6363(03)00513-3 14522424

[89] Dua MM Miyama N Azuma J Schultz GM Sho M Morser J Hyperglycemia modulates plasminogen activator inhibitor-1 expression and aortic diameter in experimental aortic aneurysm disease. Surgery (2010) 148:429–35. 10.1016/j.surg.2010.05.014 20561659 PMC2905480

[90] Bouton MC Boulaftali Y Richard B Arocas V Michel JB Jandrot-Perrus M. Emerging role of serpinE2/protease nexin-1 in hemostasis and vascular biology. Blood (2012) 119:2452–7. 10.1182/blood-2011-10-387464 22234688

[91] Bouton MC Richard B Rossignol P Philippe M Guillin MC Michel JB The serpin protease-nexin 1 is present in rat aortic smooth muscle cells and is upregulated in L-NAME hypertensive rats. Arterioscler Thromb Vasc Biol (2003) 23:142–7. 10.1161/01.ATV.0000047867.98019.2D 12524238

[92] Choi Y Chung H Jung H Couchman JR Oh ES. Syndecans as cell surface receptors: unique structure equates with functional diversity. Matrix Biol (2011) 30:93–9. 10.1016/j.matbio.2010.10.006 21062643

[93] Xian X Gopal S Couchman JR. Syndecans as receptors and organizers of the extracellular matrix. Cell Tissue Res (2010) 339:31–46. 10.1007/s00441-009-0829-3 19597846

[94] Xiao J Angsana J Wen J Smith SV Park PW Ford ML Syndecan-1 displays a protective role in aortic aneurysm formation by modulating T cell-mediated responses. Arterioscler Thromb Vasc Biol (2012) 32:386–96. 10.1161/ATVBAHA.111.242198 22173227 PMC3404811

[95] Manon-Jensen T Multhaupt HA Couchman JR. Mapping of matrix metalloproteinase cleavage sites on syndecan-1 and syndecan-4 ectodomains. FEBS J (2013) 280:2320–31. 10.1111/febs.12174 23384311

[96] Li X Herz J Monard D. Activation of ERK signaling upon alternative protease nexin-1 internalization mediated by syndecan-1. J Cell Biochem (2006) 99:936–51. 10.1002/jcb.20881 16741952

